# A30 THROUGH-THE-SCOPE CLIP WITH ANCHOR PRONGS FOR PROPHYLACTIC CLIP CLOSURE AFTER GI LESION RESECTION: A PROSPECTIVE COHORT STUDY

**DOI:** 10.1093/jcag/gwae059.030

**Published:** 2025-02-10

**Authors:** D von Renteln, D Rex, H Pohl, N Kumta, S Chan, M Ryou, N Zaheer, P H Zhou, H Inoue, J Peetermans, M Rousseau, J Mosko

**Affiliations:** Montreal, QC, Canada; Indiana University School of Medicine, Indianapolis, IN; Dartmouth Hitchcock Medical Center, Lebanon, NH; NYU Langone Health, New York, NY; Prince of Wales Hospital, Hong Kong, Hong Kong; Brigham and Women’s Hospital, Boston, MA; Asian Institute of Gastroenterology, Hyderabad, Telangana, India; Shanghai Zhongshan Hospital, Shanghai, China; Showa Daigaku Koto Toyosu Byoin, Koto-ku, Tokyo, Japan; Boston Scientific Corporation, Marlborough, MA; Boston Scientific Corporation, Marlborough, MA; StMichael’s Hospital, Toronto, ON, Canada

## Abstract

**Background:**

Endoscopic clipping following gastrointestinal (GI) lesion resection provides prophylaxis for delayed bleeding. A rotatable, anchor-pronged through-the-scope endoscopic clip (TTSC) underwent limited prospective study since commercialization in the USA in 2022.

**Aims:**

We prospectively studied 30-day delayed bleeding rates after prophylactic clip closure of lesions using the new TTSC.

**Methods:**

We conducted a multicenter, prospective cohort study of the anchor-pronged MANTIS^TM^ clip (Boston Scientific Corporation, Marlborough, Massachusetts, USA) used for prophylactic clipping after lesion resection at 10 sites in 6 countries (ClinicalTrials.gov number, NCT05653843). Outcomes were 1) complete defect closure without delayed bleeding at the original resection site, 2) rate of delayed bleeding at the original resection site, defined as a severe bleeding event requiring hospitalization, blood transfusion (>5 units), or another invasive intervention ≤30 days after study clip placement, 3) rate of serious device or procedure-related adverse events (SAEs).

**Results:**

Among 191 enrolled participants, the mean age was 65.4±12.2 (range 23-89) years, and 110 (57.6%) were male. Median maximum lesion diameter was 25.0 (range 5.0-100.0) mm. Of 199 attempted clipping procedures, 190 (95.5%) defects had complete closure without delayed bleeding at the original lesion site (Table). Delayed bleeding occurred in 1 (0.5%) patient by 30 days after the index procedure. Seven (3.7%) patients had at least one SAE, including bleeding (2 patients), perforation (1), aspiration (1), nausea (1), pneumoperitoneum (1), postpolypectomy syndrome (1). No SAEs were considered device-related.

**Conclusions:**

A rotatable, anchor-pronged TTSC showed high rates of procedural completion and sustained lesion closure, and low rates of delayed bleeding or other SAEs by 30 days after the index procedure.

Table. Main outcomes (N=199 lesions in 191 patients)

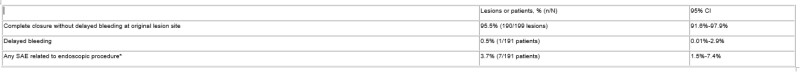

*Bleeding SAEs within 7 days were counted as failures of the primary efficacy endpoint.

**Funding Agencies:**

Boston Scientific Corporation

